# Possible Cis-acting signal that could be involved in the localization of different mRNAs in neuronal axons

**DOI:** 10.1186/1742-4682-2-33

**Published:** 2005-08-24

**Authors:** Gonzalo E Aranda-Abreu, Ma Elena Hernández, Abraham Soto, Jorge Manzo

**Affiliations:** 1 Instituto de Neuroetología, Universidad Veracruzana, Av. Dos Vistas S/N, km 2.5 Carr. Xalapa-Veracruz. Col. Industrial-Animas. C.P. 91190. Xalapa, Ver. México

**Keywords:** mRNA, U-rich region, axon

## Abstract

**Background:**

Messenger RNA (mRNA) comprises three major parts: a 5'-UTR (UnTranslated Region), a coding region, and a 3'-UTR. The 3'-UTR contains signal sequences involved in polyadenylation, degradation and localization/stabilization processes. Some sequences in the 3'-UTR are involved in the localization of mRNAs in (e.g.) neurons, epithelial cells, oocytes and early embryos, but such localization has been most thoroughly studied in neurons. Neuronal polarity is maintained by the microtubules (MTs) found along both dendrites and axon and is partially influenced by sub-cellular mRNA localization. A widely studied mRNA is that for Tau protein, which is located in the axon hillock and growth cone; its localization depends on the well-characterized cis-acting signal (U-rich region) in the 3'-UTR.

**Methods:**

We compared the cis-acting signal of Tau with mRNAs in the axonal regions of neurons using the ClustalW program for alignment of sequences and the Mfold program for analysis of secondary structures.

**Results:**

We found that at least 3 out of 12 mRNA analyzed (GRP75, cofilin and synuclein) have a sequence similar to the cis-acting signal of Tau in the 3'-UTR. This could indicate that these messengers are localized specifically in the axon. The Mfold program showed that these mRNAs have a similar "bubble" structure in the putative sequence signal.

**Conclusion:**

Hence, we suggest that a U-rich sequence in the 3'-UTR region of the mRNA could act as a signal for its localization in the axon in neuronal cells. Sequences homologous to the DTE sequence of BC1 mRNA could direct the messenger to the dendrites. Messengers with homologues of both types of sequence, e.g. β-actin, might be located in both dendrites and axon.

## Background

A messenger RNA (mRNA) comprises three major parts, a 5'-UTR (UnTranslated Region), a coding region and a 3'-UTR. The 3'-UTR contains signal sequences involved in polyadenylation, degradation and localization/stabilization processes. Many studies have shown that certain sequences in the 3'-UTR are involved in localizing the mRNAs in different cells such as neurons, epithelial cells, oocytes and early embryos [1,2]. Such localization has been studied exhaustively in neurons. Neurons are polar cells, with dendrites and axon; dendrites receive information and the axon is specialized to transmit this information to the next neuron [3]. The maintenance of neuronal polarity depends on the microtubules (MTs) [3-6], which are found along both axon and dendrites, and is partially determined by subcellular mRNA localization. The mechanism responsible for creating the polarity involves synergistic controls of translation, stabilization and association with elements of the cytoskeleton.

The mRNA of tau has been studied in detail [7]. Tau is located in the axon hillock and growth cone; the well-characterized cis-acting signal (U-rich region) located in the 3'-UTR of its mRNA is responsible for its localization [8]. HuD protein interacts with this U-rich sequence to form a mRNA-protein complex that is transported toward the axon (axon hillock and growth cone) by interacting with KIF3A, a kinesin responsible for anterograde movement [9-11].

Recently, many mRNAs have been shown to be located in neuronal axons: β-actin, tropomyosin 3 (Tpm3), cofilin, vimentin, immunoglobulin heavy chain biding protein (Bip), heat shock protein 60 (HSP60), heat shock protein 70 (HSP70), heat shock protein 90 (HSP90), glucose regulated protein (grp75) and synuclein [12]. The objective of this paper is to determine, using bioinformatics tools, whether there is a cis-acting signal in all the mRNAs that are transported to the axon and whether this putative signal is similar to the U-rich region in the 3'-UTR of tau mRNA.

## Results

The 3'-UTR of tau mRNA contains 3884 bases; the U-rich region (in bold) is responsible for the localization of this mRNA in the axon hillock and growth cone.

UCAGGCCCCUGGGGCCGUCACUGAUCAUGGAGAGAAGAGAGAGUGAGAGUGUGGAAAAAAAAAAAAAAAGAAUGACCUGGCCCCUCACCCUCUGCCCUCCCCGCUGCUCCUCAUAGACAG GCUGACCAGCUUGUCACCUAACCUGCUUUUGUGGCUCGGGUUUGGCUCGGGACUUCAAAAUCAGUGAUGGGAAAAAGUAAAUUUCAUCUUUCCAAAUUGAUUUGUGGGCUAGUAAUAAAA UAUUUUUAAGGAAGGAAAAAAAAAACACGUAAAACCAUGGCCAAACAAAACCCAACAUUUCCUUGGCAAUUGUUAUUGACCCCGCCCCCCCCUCUGAGUUUUAGAGGGUGAAGGAGGCUU UGGAUAGAGGCUGCUUCUGGGGAUUGGCUGAGGGACUAGGGCAACUAAUUGCCCACAGCCCCAUCUUAGGGGCAUCAGGGACAGCGGCAGACAUGAAAGACUUGGGACUUGGUGUGUUUG UGGAGCCGUAAGGCGUAUGUUAACUUUGUGUGGGUUUGAGGGAGGACUGUGAUAGUGAAGGCUGAGAGAUGGGUGGGCUGGGAGUCAGAGGAGAGAGGUGAGGAAGACAGGUUGGGAGAG GGGGCAUUGCGUCCUUGCCAAGGAGCUUGGGAAGCACAGGUAGCCCUGGCUGCAGCAGUCUUAGCUAGCACAGAUGCCUGCCUGAGAAAGCACAGUGGGGUACAGUGGGUGUGUGUGCCC CUUCUGAAGGGCAGCCCAUGGGAGAAGGGGUAUUGGGCAGAAGGAAGGUAGGCCCCAGAAGGUGGCACCUUGUAGAUUGGUUCUCUGAAGGCUGACCUUGCCAUCCCAGGGCACUGCUCC CACCCUCCAGGAGGAGGUCUGAGCUGAGGAGCUUCCUUUUCGAUCUCACAGGAAAACCUGUGUUACUGAGUUCUGAAGUUUGGAACUACAGCCAUGAUUUUGGCCACCAUACAGACCUGG GACUUUAGGGCUAACCAGUUCUUUGUAAGGACUUGUGCCUCUUGCGGGAACAUCUGCCUGUUCUCAAGCCUGGUCCUCUGGCACUUCUGCAGUGGUGAGGGAUGGGGGUGGUAUUCUGGG AUGUGGGUCCCAGGCCUCCCAUCCCUCGCACAGCCACUGUAUCCCCUCUACCUGUCCUAUCAUGCCCACGUCUGCCACGAGAGCCAGUCACUGCCGUCCGUACAUCACGUCUCACCGUCC UGAGUGCCCAGCCUCCCAAGCCCAAUCCCUGGACCCCUGGGUAGUUAUGGCCAAUCUGCUCUACACUAGGGGUUGGAGUCCAGGGAAGGCAAAGAUUUGGGCCUUGGUCUCUAGUCCUAC GUUGCCAGAAUCCAACCAGUGUGCCUCCCACAAGGAACCUUACAACCUUGUUUGGUUUGCUCCAUCAGGCGUUUGGCGCCAUCGUGGAUGGAGUCCGUGUGUGCCUGGAGAUUACCCUGG ACACCUCUGC**UUUUUUUUUUUUU**ACUUUAGCGGUUGCCUCCUAGGCCUGACUCCUUCCCAUGUUGAACUGGAGGCAGCCAAGUUAGGUGUCAAUGUCCUGGCAUCAGUAUGAACAGUC AGUAGUCCCAGGGCAGGGCCACACUUCUCCCAUCUUCUGCUUCCACCCCAGCUUGUGAUUGCUAGCCUCCCAGAGCUCAGCCGCCAUUAAGUCCCCAUGCACGUAAUCAGCCCUUCCAUA CCCCAAUUUGGGGAACAUACCCCUUGAUUGAAAUGUUUUCCCUCCAGUCCUAUGGAAGCGGUGCUGCCUGCCUGCUGGAGCAGCCAGCCAUCUCAGAGACGCAGCCCUUUCUCUCCUGUC CGCACCCUGCUGCGCUGUAGUCGGAUUCGUCUGUUUGUCUGGGUUCACCAGAGUGACUAUGAUAGUGAAAAGAAAAAGAAAAAGAAAAAAGAAAAAAGAAAAAAAAAAAAGGACGCAUGU UAUCUUGAAAUAUUUGUCAAAAGGUUGUAGCCCACCGCAGGGAUUGGAGGGCCUGAUAUUCCUUGUCUUCUUCGUGACUUAGGUCCAGGCCGGUCGAGUGCUACCCUGCUGGACAUCCCA UGUUUUGAAGGGUUUCUUCUUCAUCUGGGACCCCUGCAGACACUGGAUUGUGACAUUGGAGGUCUAUACAUUGGCCAAGGCUGAAGCACAGGACCCGUUAGAGGCAGCAGGCUCCGACUG UCAGGGAGAGCUUGUGGCUGGCCUGUUUCUCUGAGUGAAGAUGGUCCUCUCUAAUCACAACUUCAAGUCCCACAGCAGCCCUGGCAGACAUCUAAGAACUCCUGCAUCACAAGAGAAAAG GACACUAGUACCAGCAGGGAGAGCUGUGGCCCUAGAAAUUCCAUGACUCUCCACUACUAUCCGUGGGUCCUUUCCAAGCCUUGCCUCGUCACCAAGGGCUUGGGAUGGACUGCCCCACUG AUGAAAGGGACAUCUUUGGAGACCCCCUUGGUUUCCAAGGCGUCAGCCCCCUGACCUUGCAUGACCUCCUACAGCUGAAGGAUGAGGCCUUUAAAGAUUAGGAACCUCAGGCCCAGGUCG GCCACUUUGGGCUUGGGUACAGUUAGGGACGAUGCGGUAGAAGGAGGUGGCCAACCUUUCCAUAUAAGAGUUCUGUGUGCCCAGAGCUACCCUAUUGUGAGCUCCCCACUGCUGAUGGAC UUUAGCUGUCCUUAGAAGUGAAGAGUCCAACGGAGGAAAAGGAAGUGUGGUUUGAUGGUCUGUGGUCCCUUCAUCAUGGUUACCUGUUGUGGUUUUCUCUGUAUACCCCCAUUUACCCAU CCUGCAGUUCCUGUCCUUGAAUAGGGGUGGGGGUACUCUGCCAUAUCUCUUGUAGGCAGUCAGCCCCCAAGUCAUAGUUUGGAGUGAUCUGGUCAGUGCUAAUAGGCAGUUUACAAGGAA UUCUGGCUUGUUACUUCAGUGAGGACAAUCCCCCAAGGCCCUGGCACCUGUCCUGUCUUUCCAUGGCUCUCCACUGCAGAGCCAAUGUCUUUGGGUGGGCUAGAUAGGGUGUACAAUUUG CCUGGAACCUCCAAGCUCUUAAUCCACUUUAUCAAUAGUUCCAUUUAAAUUGACUUCAAUAUAAGAGUGUAUCCAUUUGAGAUUGCUUGUGUUGUGGGGUAAAGGGGGGAGGAGGAACAU GUUAAGAUAAUUGACAUGGGCAAGGGGAAGUCUUGAAGUGUAGCAGUUAAACCAUCUUGUAGCCCCAUUCAUGAUGUUGACCACUUGCUAGAGAGAAGAGGUGCCAUAAGGCUAGAACCU AGAGGCUUGGCUGUCCACCAACAGGCAGGCUUUUGCAAGGCAGAGGCAGCCAGCUAGGUCCCUGACUUCCCAGCCAGGUGCAGCUCUAAGAACUGCUCUUGCCUGCUGCCUUCUUGUGGU GUCCAGAGCCCACAGCCAAUGCCUCCUCAAAACCCUGGCUUCCUUCCUUCUAAUCCACUGGCACAUCAGCAUCACCUCCGGAUUGACUUCAGAUCCACAGCCUACACUACUAGCAGUGGG UAAGACCACUUCCUUUGUCCUUGUCUGUUCUCCAGAAAAGUGGGCAUGGAGGCGGUGUUAAUAACUAUAGGUCUGUGGCUUUAUGAGCCUUCAAACUUCUCUCUAGCUUCUGAAAGGGUU ACUUUUGGGCAGUAUUGCAGUCUCACCCUCCGAUGGCUGUAGCCUGUGCAGUUGCUGUACUGGGCAUGAUCUCCAGUGCUUGCAAGUCCCAUGAUUUCUUUGGUGUUUUGAGGGUGGGGG GAGGGACAUGAAUCAUCUUAGCUUAGCUUCCUGUCUGUGAAUGUCCAUAUAGUGUACUGUGUUUUAACAAACGAUUUACACUGACUGUUGCUGUACAAGUGAAUUUGGAAAUAAAGUUAU UACUCUGAUUAAACAAAAAAAAAAAAAAAAAAAAAAAAAAAAAAAA

This cis-acting signal of tau was compared base by base with the other afore mentioned mRNAs using simple alignment. We also made comparisons with another sequence that is specific for the localization of BC1 mRNA in dendrites, the Dendritic Target Element (DTE) [13].

In the β-actin mRNA of chicken a cis-acting signal "zipcode" has been described; a zipcode binding protein binds to this sequence and this is a prerequisite for the localization of the mRNA. The sequence is a tandem repeat of an ACACCCACACCC motif. The mRNA of β-actin has been located in the axon of the neuron and in dendritic spines [12,14]. β-actin mRNA has a sequence closely similar to the tau signal in the first part of its 3'-UTR, but there is also another sequence that could participate in its localization in dendrites; this sequence is very similar to the DTE [13]. The protein tropomyosin 3 has been located in the growth cone of the neuron; its mRNA has also been detected in axons during development [15]. Both tropomyosin 3 and β-actin form parts of the cytoskeleton. No well-defined sequence signal that could be involved in the specific localization of these messengers in the axon (tpm3 and β-actin) has been identified, so it is likely that β-actin and tropomyosin are not exclusive to the axon and could be also found in dendrites.



Cofilin is a cytoskeleton modulating protein; it is also known as actin depolymerizing factor (ADF). The potential role of cofilin is to modulate the changes of actin organization that accompany neurite initiation, axonogenesis and growth cone guidance [16]. The possible signal sequence found in the 3'-UTR of cofilin mRNA is very similar to that of tau; they share a U-rich region, which indicates that this messenger might be transported to the growth cone of the developing neuron. However, a possible DTE sequence is also present, located upstream of the U-signal.



Vimentin has been located by RT-PCR in the axons of dorsal root ganglia (DRG) neurons. A possible sequence signal in vimentin mRNA shares some U with tau but also contains more purines, which might indicate that the protein is not exclusive to the axonal region [12].



Bip is a protein that binds to the immunoglobin heavy chains in pre-β cells. Its mRNA shares some U with the tau sequence; nevertheless, its sequence suggests that this mRNa, like vimentin, is probably not exclusive to the axon [17].



The heat shock proteins and grp75 messengers have similarities with the tau sequence, but once again they are probably not exclusive to the axon. They could interact with other proteins in different parts of the cell.







Synuclein is a soluble unfolded protein that can aggregate into insoluble fibrils under several pathological conditions including Parkinson's and Alzheimer's diseases [12]. The possible cis-acting signal of the mRNA for this protein is very similar to the tau signal, with only a single U to C substitution, suggesting that the synuclein messenger may be transported to the axon by a similar mechanism to the tau messenger and that aggregation and precipitation of the synuclein protein within the axon contributes to neurodegenerative disease.



These analyses carried out by alignment allowed us to show that the cis-acting signals of the mRNAs examined have some homology with that of the tau messenger.



**Figure 1:**
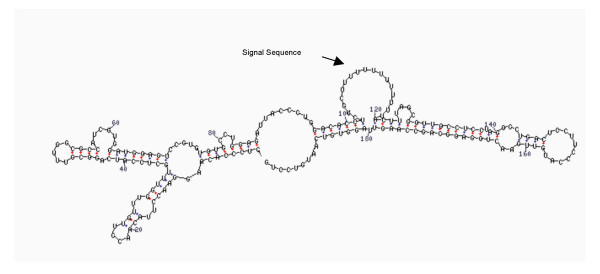
RNA secondary structure of 3'-UTR of tau mRNA. The arrow indicates the "bubble" where the HuD binds to stabilize the messenger.

**Figure 2:**
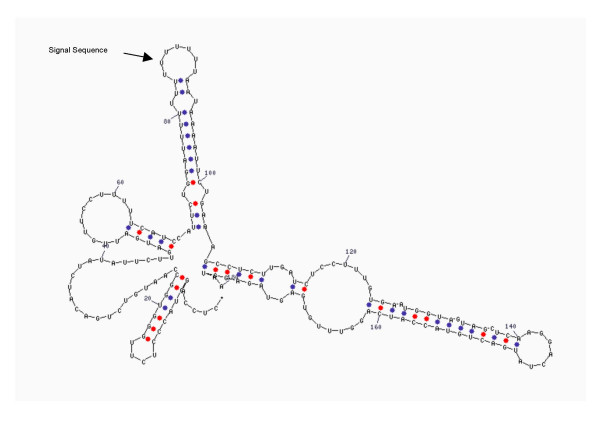
RNA secondary structure of 3'-UTR of GRP75 mRNA. The arrow indicates the U-rich signal sequence that could be involved in the localization of the messenger.

**Figure 3:**
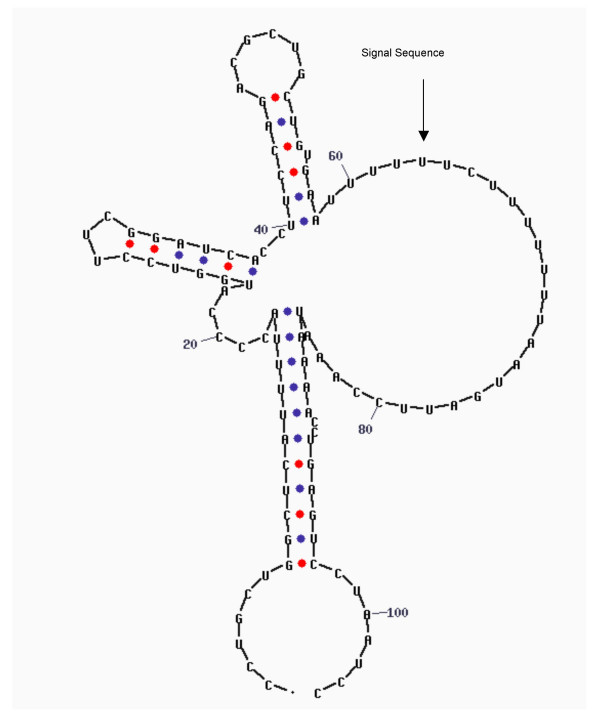
RNA secondary structure of 3'-UTR of synuclein mRNA. The arrow indicates the U-rich signal sequence that could be involved in the localization of the messenger.

**Figure 4:**
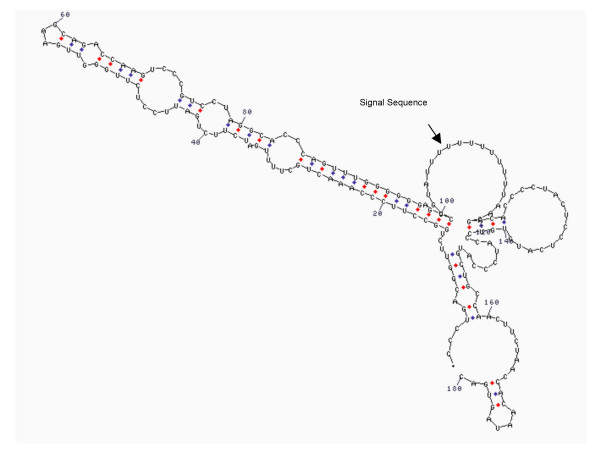
RNA secondary structure of 3'-UTR of cofilin mRNA. The arrow indicates the U-rich signal sequence that could be involved in the localization of the messenger.

**Figure 5:**
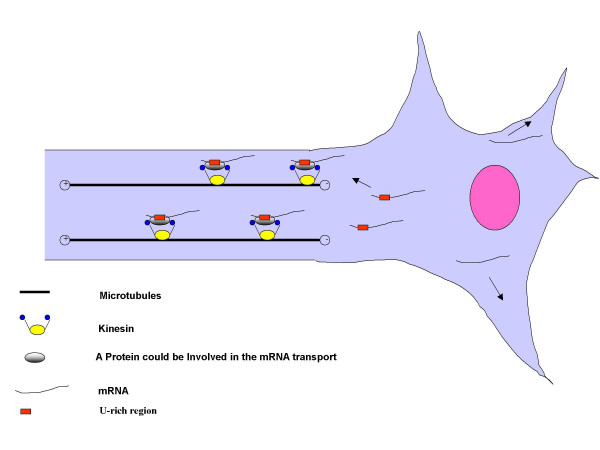
A model of the 3'-UTR/U-rich region by virtue of which the mRNA could be transported toward the axon. The mRNAs that contain a U-rich sequence in the 3'-UTR are candidates for transport toward the axon. The model suggests that an mRNA binding protein intreacts with the signal-sequence forming a putative complex that is anchored to a kinesin protein. The mRNAs that do not contain such a U-sequence might remain in the cell body or to migrate towards the dendrites.

The highest homology scores are:



The secondary structures of these four mRNAs, which showed the closest homologies to the tau sequence, were analyzed using the program Mfold (Figs. 1, 2, 3, 4). Fig. 5 shows a model of U-rich mRNAs that could be transported to the axon. The results show that the secondary structures of synuclein and cofilin mRNAs are very similar to that of the tau messenger, and the cis-acting signal sequence is inside the "bubble" according to the Mfold program. This indicates to us that both the signal sequence and the secondary structure could be determining factors in the location of these messengers in the axon region.

## Discussion

The first messenger to be analyzed in the 3'-UTR with respect to its localization/stabilization was tau [9,10]. The U-rich region in its signal sequence enables the formation of a complex with HuD, a prerequisite for transport to the axon, and increases the stability of the mRNA. The basic function of tau protein is to stabilize the microtubules; it prevents depolymerization and consequent loss of neuronal polarity. Recently, several other messengers have been shown by RT-PCR to be localized in neuronal axon of the neuron, but the possibility that these mRNAs are also located in the dendrites has not been excluded.

### GRP75

Glucose regulated protein 75 (GRP75) is an important molecular chaperon belonging to the heat shock protein (HSP) family. It is highly expressed in conditions of glucose deprivation of glucose. Its messenger was located in the axon and it has a U-rich region. It might not be confined exclusively to the axon because this protein responds to a metabolic stress [18].

### Synuclein

Alfa-synuclein is involved in neurodegenerative diseases and its presence has been observed in the pre-synaptic and nuclear compartments, though the location in the nucleus has not been well documented. The synuclein messenger possesses a U-rich region; nevertheless a C interrupts the potential signal sequence. When it is wrongly folded, this protein may aggregate in the cell forming fibrils, typical of Alzheimer's and Parkinson's diseases. The aggregation of synuclein is similar to tau in Alzheimer patients, which could indicate similar intracellular behavior by both proteins [19].

### Cofilin

The 3'-UTR of the cofilin messenger has a U-rich region very similar to the signal sequence of tau, which on the face of it suggests that it might be located exclusively in the axon. Nevertheless, recent studies demonstrate that it participates in the shrinkage of dendritic spines associated with the long-term depression of hippocampal synapses, suggesting that it is also found in dendrites. Moreover, it is involved in neuronal development, axogenesis, guidance of the growth cone and dendrite formation. Although the cofilin messenger is present in axons, the possible participation of the protein in events related to the unplugging of synapses because of its association with actin further suggests that it is not confined to the axon but also occurs in the dendrites [16].

### β-actin

The 3'-UTR of the β-actin messenger is very short and shows low homology when aligned with the tau cis-acting signal. However, when it was aligned with the dendritic target element, it showed better homology. The β-actin messenger was shown to possess a zipcode that leads it towards the dendrites instead of the axon [14].

### HSP70 and HSP90

Molecular chaperones and their functions in protein folding have been implicated in several neurodegenerative conditions, including Parkinson's and Huntington's diseases, which are characterized by accumulation of protein aggregates (e.g. α-synuclein and huntingtin, respectively). These aggregates have been shown in various experimental systems to respond to changes in levels of molecular chaperones, suggesting the possibility of therapeutic intervention and a role for chaperones in disease pathogenesis. It remains unclear whether chaperones also play a role in Alzheimer's disease, a neurodegenerative disorder characterized by β-amyloid and tau protein aggregates. In various cellular models, increased levels of Hsp70 and Hsp90 promote tau solubility and tau binding to microtubules, reduce insoluble tau and cause reduced tau phosphorylation. Conversely, lowered levels of Hsp70 and Hsp90 result in the opposite effects. A direct association between the chaperones and tau protein has been demonstrated. Many results suggest that the up-regulation of molecular chaperones may suppress the formation of neurofibrillary tangles by partitioning tau into a productive folding pathway and thereby preventing tau aggregation [20]. When we compared the 3'-UTRs of the messengers for these chaperones, they showed some homology with the cis-acting signal of tau because each possesses a U-rich region, which could indicate that they are found in axons.

## The model

On the basis of the results we suggested a model for mRNA localization in the axon (Fig. 5).

The mRNAs containing the U-rich region could be complexed with a protein responsible for transport toward the axon, just as HuD complexes with and stabilizes the tau messenger [9]. HuD itself has the capacity to bind to different mRNAs such as GAP-43 [21], neuroserpin [22], acetylcholinesterase [23] and c-myc [24], so it might interact with other messengers with a U-rich signal, stabilizing the messenger and facilitating transport to the axon. The motor protein that translocates the complex along the axonal microtubules could belong to the kinesin family, by analogy with the translocation of the tau messenger by the kinesin KIF3A [11]. When the complex reaches the correct destination, the mRNA is translated. mRNAs that lack the U-rich sequence presumably go to another cellular compartment in the neuron; those with DTE-like signals might preferentially accumulate in the dendrites.

The mechanisms determining whether a messenger such as β-actin is transported preferentially to the axon or the dendrites are poorly understood. The existence of two potentially conflicting location signals in the 3'-UTR (one U-rich and tau-like, the other DTE-like) raises questions about how the final destination of such mRNAs is determined within the neuron.

## Conclusion

In the 3'-UTRs of some mRNAs in neurons there are cis-acting signals that direct mRNAs such as tau and GAP-43 to the axon. In general, these signals are rich in uridine and do not contain guanidine. Comparison of the Dendritic Target Element (DTE) with the 3'-UTRs of several axon-located messengers showed some homology in a specific region of the 3'-UTR. Most of the 3'-UTRs studied possess homologies with the signals involved in the localization of mRNAs in axons and dendrites. This might explain why as much β-actin is present in dendrites as in axons, though the distribution mechanisms in such cases are not understood. In addition, we found a DTE homology in the 3'-UTR of HSP70 and 90. The significance of this is not clear; some messengers are transported towards the axon or towards the dendrites as required.

A sequence homologous to DTE in tau occurs near the end of the 3'-UTR, next to the polyadenylation site, which indicates that only the axon signal sequence (not the dendrite signal sequence) is functional, because mRNA degradation starts at the poly(A) site. The 3'-UTR of MAP2 [25] possesses no homology with the axon signal sequence, suggesting that as many tau as MAP2 mRNAs are transported exclusively to their respective regions inside the neuron.

Very U-rich sequences in the 3'-UTR might be signals that direct some mRNAs exclusively to the axon. If we understand which signals/sequences the neuronal cell uses for the correct location of its mRNAs, it might become possible to determine which factors lead to mislocalization of messengers and of proteins, as has recently been suggested in relation to certain neurodegenerative diseases such as Alzheimer's.

## Methods

All the mRNAs analyzed in this study belong to the *Rattus norvegicus *genome and were located using the following GeneBank accession numbers. β-actin; NM_031144, tropomyosin 3 (Tpm3); NM_057208, cofilin; NM_017147, vimentin; NM_031140, immunoglobulin heavy chain biding protein (Bip); M14050, heat shock protein 60 (HSP60); X53585, heat shock protein 70 (HSP70); L16764, heat shock protein 90 (HSP90); S45392, glucose regulated protein (grp75); s78556, synuclein; NM_031688; NM_057114 and NM_053576 and tau; X79321. The 3'-UTRs of the mRNAs were analyzed using the program ClustalW [26], and the secondary structures were generated by the Mfold program [27].

## Competing interests

The author(s) declare that they have no competing interests.
